# Preclinical Pharmacokinetics and Biodistribution of LR004, a Novel Antiepidermal Growth Factor Receptor Monoclonal Antibody

**DOI:** 10.3390/molecules29020545

**Published:** 2024-01-22

**Authors:** Ying Zheng, Guifang Dou, Shuchen Liu, Zhiyun Meng, Eric I. Tsao, Gang Yu, Xiaoxia Zhu, Ruolan Gu, Zhuona Wu, Yunbo Sun, Peng Han, Hui Gan

**Affiliations:** 1 Department of Pharmaceutical Sciences, Beijing Institute of Radiation Medicine, Beijing 100850, China; yzheng@icmm.ac.cn (Y.Z.); dougf@bmi.ac.cn (G.D.); liusc118@163.com (S.L.); mengzhiyun@vip.163.com (Z.M.); 13681022512@163.com (X.Z.); gurl311@126.com (R.G.); wznphd@126.com (Z.W.); sunyunbo0919@126.com (Y.S.); 15901135949@163.com (P.H.); 2 Artemisinin Research Center, China Academy of Chinese Medical Sciences, Beijing 100700, China; 3 Synermore Biologics Co., Ltd., Suzhou 215000, China; etsao@synermore.com; 4 State Key Laboratory of Toxicology and Medical Countermeasures, Beijing Institute of Pharmacology and Toxicology, Beijing 100850, China; yg1st@163.com

**Keywords:** monoclonal antibody, EGFR, tumor-targeting, pharmacokinetics, radionuclide-based biodistribution, nanoSPECT/CT

## Abstract

LR004 is a novel chimeric (human/mouse) monoclonal antibody developed for the treatment of advanced colorectal carcinoma with detectable epidermal growth factor receptor (EGFR) expression. We aimed to investigate the preclinical pharmacokinetics (PK) and in vivo biodistribution of LR004. The PK profiles of LR004 were initially established in rhesus monkeys. Subsequently, ^125^I radionuclide-labeled LR004 was developed and the biodistribution, autoradiography, and NanoSPECT/CT of ^125^I-LR004 in xenograft mice bearing A431 tumors were examined. The PK data revealed a prolonged half-life and nonlinear PK characteristics of LR004 within the dose range of 6–54 mg/kg. The radiochemical purity of ^125^I-LR004 was approximately 98.54%, and iodination of LR004 did not affect its specific binding activity to the EGFR antigen. In a classical biodistribution study, ^125^I-LR004 exhibited higher uptake in highly perfused organs than in poorly perfused organs. Prolonged retention properties of ^125^I-LR004 in tumors were observed at 4 and 10 days. Autoradiography and NanoSPECT/CT confirmed the sustained retention of ^125^I-LR004 at the tumor site in xenograft mice. These findings demonstrated the adequate tumor targeting capabilities of ^125^I-LR004 in EGFR-positive tumors, which may improve dosing strategies and future drug development.

## 1. Introduction

Colorectal cancer (CRC) is one of the most lethal and common cancers worldwide. Approximately 25% of patients are diagnosed at an advanced stage with metastasis, whereas an additional 20% of the cases may subsequently develop metachronous metastasis [1,2]. The 5-year survival rate of patients with metastatic CRC is 65%; thus, the development of new therapeutic approaches for metastatic CRC is necessary [3,4].

The epidermal growth factor receptor (EGFR) is a receptor tyrosine kinase that is commonly upregulated in various cancers, such as non-small–cell lung cancer, metastatic colorectal cancer, head and neck cancer, and breast cancer. EGFR plays a crucial role in the proliferation, survival, and transformation of cancer cells and has emerged as an important molecule for enhanced tumor targeting. Because of its overexpression in 25–82% of colorectal carcinomas, EGFR has been identified as an invaluable therapeutic target for colorectal carcinoma metastases [5,6]. Many EGFR-targeted agents have been developed to date, including small molecule tyrosine kinase inhibitors such as gefitinib, erlotinib, and lapatinib, as well as monoclonal antibodies such as cetuximab and panitumumab. Compared with traditional cytotoxic drugs, monoclonal antibodies exhibit better selectivity and fewer adverse side effects. Cetuximab (Erbitux^®^), a recombinant human/mouse chimeric anti-EGFR monoclonal antibody, approved by the FDA in 2004, was the first monoclonal antibody targeting EGFR for patients with EGFR-expressing metastatic colorectal cancer after failure of both irinotecan- and oxaliplatin-based regimens or for those who are intolerant of irinotecan-containing regimens [7,8]. However, recent studies have shown that KRAS mutations are detected in 36–46% of colorectal cancer cases, rendering cetuximab ineffective for the treatment of such patients [9,10,11]. Despite the expiration of the patent in Europe in 2014 and projected annual sales of approximately USD 1.681 million in 2022, Erbitux^®^ has not yet faced any approved biosimilar challenges in the United States or in Europe [12]. The exorbitant costs associated with multiple treatment courses underscore the pressing need for novel anti-EGFR agents.

LR004 is a novel antibody molecule that shares several key characteristics with cetuximab, including its mouse-human chimeric composition structure, its targeting of a domain of EGFR, its relatively long half-life, and its activity in colorectal cancer. Unlike cetuximab, which has murine glycan structures, LR004 has only a human glycan structure. This characteristic diminishes the antibody’s heterogeneity and enhances its suitability for future clinical applications in humans. LR004 has demonstrated antitumor activity in various tumor models, including epidermoid carcinoma (A431), colon cancer (GEO), and breast cancer (MDA-MB-468) [13]; however, comprehensive preclinical pharmacokinetic (PK) and biodistribution profiles for LR004 have not been established. These profiles are necessary for dosage formulations and clinical studies as a novel antibody agent.

Here, we conducted a preclinical investigation of the PK properties and in vivo biodistribution profile of LR004. Initially, we determined the affinity and kinetics of the LR004 antibody toward the EGFR antigen using a BIAcore X100 capture assay. Next, we examined the PK of LR004 at various doses by intravenous infusion in rhesus monkeys. Furthermore, we labeled LR004 with the ^125^I isotope and used three methods to examine the biodistribution of ^125^I-LR004 within tumor-bearing BALB/c nude mice models, with a particular emphasis on its distribution within tumor tissues. Among these methods, the radionuclide-based biodistribution study represents a conventional and straightforward approach that provides detailed data on the accumulation of radiolabeled antibodies in excised tissues, which offers more accurate prognostic and predictive information. The combination of antibody labeling and bio-imaging techniques plays an important role in establishing the in vivo distribution characteristics of novel antibody molecules. This approach has been increasingly adopted because of its ability to provide a comprehensive understanding of tumor biology as well as its specific binding affinity toward the intended targets [14,15,16]. Therefore, we applied autoradiography and single photon emission computer tomography (SPECT) imaging technology to obtain direct visual verification of the distribution properties of ^125^I-LR004.

## 2. Results

### 2.1. Antibody Affinity and Kinetics Assay

**Figure 1:**
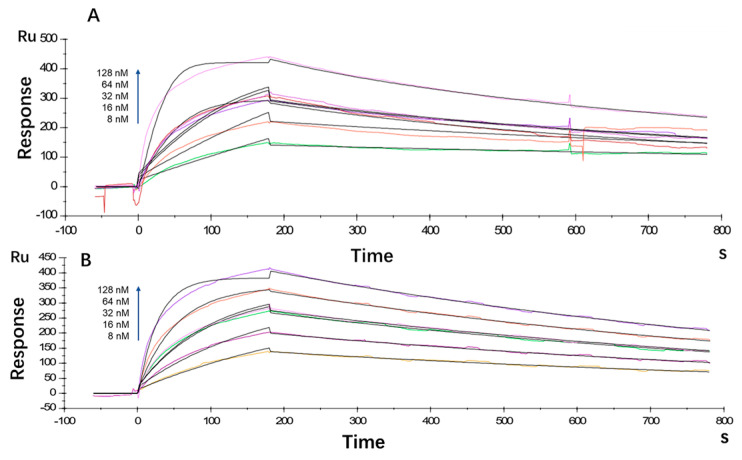
Kinetic analysis/affinity test fitting curves from BIAcore analysis. (**A**) Kinetic analysis/affinity test fitting curve of LR004; (**B**) Kinetic analysis/affinity test fitting curve of cetuximab.

The antibody affinity and kinetics assay using the BIAcore X100 System showed that the equilibrium dissociation constant (KD) for LR004 was 2.80 × 10^−9^ M, with a ka value of 5.26 × 10^5^ 1/Ms and a kd value of 1.47 × 10^−3^ 1/s (Figure 1A). Cetuximab exhibited a KD of 3.88 × 10^−9^ M, ka of 2.86 × 10^5^ 1/Ms, and kd of 1.11 × 10^−3^ 1/s (Figure 1B). Thus, the affinity between LR004 and EGFR showed moderate strength comparable with that of the commercially available cetuximab.

### 2.2. PK Profiles of LR004 in Rhesus Monkeys

**Figure 2:**
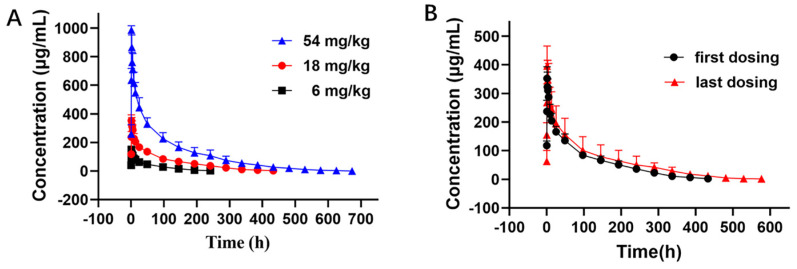
Serum concentration–time curve of LR004 in rhesus monkeys. (**A**) Serum concentration profiles of LR004 following a single intravenous infusion to rhesus monkeys at doses of 6, 18, and 54 mg/kg (mean ± SD, n = 6); (**B**) Serum concentration profiles of LR004 at a dose of 18 mg/kg for first/last dosing (mean ± SD, n = 6).

The PK curves of LR004 following a single intravenous infusion are shown in Figure 2A. The PK parameters in Table 1 indicate that the elimination half-lives (t_1/2_) at doses of 6, 18, and 54 mg/kg were 51.60 ± 8.36, 104.62 ± 13.42, and 130.72 ± 39.57 h, respectively, and the t_1/2_ gradually extended with increasing doses. The respective AUC_last_ were 6900.24 ± 1243.11, 25,398.82 ± 4774.38, and 72,301.13 ± 16,411.89 mg·h/L. The AUC rate was 1:3.68:10.48, which was consistent with the dose rate of 1:3:9. The MRT_last_ and Vd also significantly increased with increasing doses. These parameters suggest that LR004 exhibits nonlinear PK characteristics within a dose range of 6–54 mg/kg in rhesus monkeys. As shown in Figure 2B, after four consecutive intravenous infusions of LR004 at a dose of 18 mg/kg, there were no statistically significant differences between the first and last dosing profiles, indicating negligible plasma accumulation of LR004 following continuous administration. The PK parameters had no statistical differences among different sexes (*p* > 0.05).

### 2.3. Radiochemical Purity Identification

**Figure 3:**
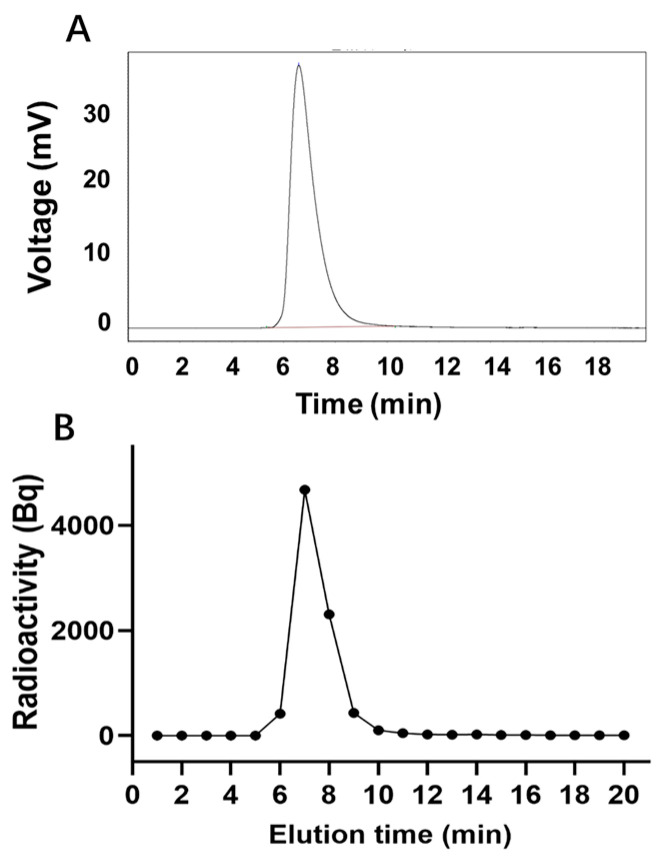
Radiochemical purity determination. (**A**) SHPLC profiles of unlabeled LR004; (**B**) Radiochemical purity profiles of ^125^I-LR004.

The peak time of unlabeled LR004 was 6.59 min (Figure 3A), whereas the peak time of ^125^I-LR004 was 6.5 min (Figure 3B), consistent with that of unlabeled LR004. This suggests that the SHPLC elution behavior of LR004 was unchanged before and after ^125^I-labeling. The radiochemical purity of ^125^I-LR004 indicated that it was successfully synthesized as evidenced by the radioactive peak area ratio. The average of the three measurements was 98.54%.

### 2.4. In Vitro Inhibition of Tumor Cells

**Figure 4:**
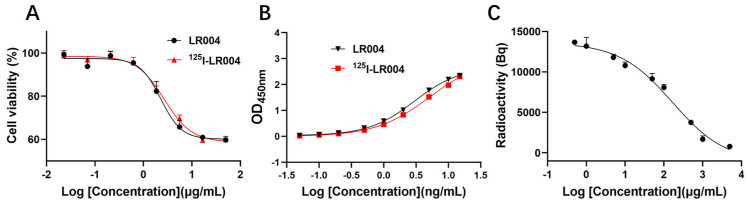
Inhibition and binding activities of LR004 before and after radiolabeling. (**A**) Curves showing the inhibition of proliferation by unlabeled LR004 and ^125^I-LR004 in MDA-MB-468 cells, as detected by CCK-8. The concentration of LR004 and ^125^I-LR004 were 0.023, 0.07, 0.21, 0.62, 1.85, 5.56, 16.67, and 50 μg/mL (left to right on the X-axis); (**B**) Comparison of antigen binding activities between unlabeled LR004 and ^125^I-LR004, as determined via ELISA. The concentration of LR004 and ^125^I-LR004 were 0.05, 0.1, 0.2, 0.5, 1, 2, 5, 10, and 15 ng/mL (left to right on the X-axis); (**C**) Competitive binding of ^125^I-LR004 and unlabeled LR004 to MDA-MB-468 cells, as quantified via γ counter. The concentrations of unlabeled LR004 were 0.5, 1, 5, 10, 50, 100, 500, 1000, 5000 μg/mL (left to right on the X-axis).

As shown in Figure 4A, the curves of the inhibition of proliferation by unlabeled LR004 and ^125^I-radiolabeled LR004 were similar, and the EC_50_ values were 2.278 and 2.671 µg/mL, respectively. Thus, iodination of LR004 did not affect its inhibitory effect on tumor cells.

### 2.5. In Vitro Binding Activities to the EGFR Antigen

The antigen binding curves of a series of ^125^I-LR004 concentrations were identical to those of non-labeled LR004, as determined via ELISA (Figure 4B). The binding activities of the two groups to the EGFR antigen were in good agreement.

### 2.6. Specific Binding Affinity toward MDA-MB-468 Cells

As shown in Figure 4C, the concentration of ^125^I-labeled LR004 was fixed at 50 µg/mL. When the concentration of unlabeled LR004 was varied (0.5, 1, 5, 10, 50, 100, 500, 1000, and 5000 μg/mL), cell-bound radioactivity analysis demonstrated that unlabeled LR004 exhibited a dose-dependent competition with ^125^I-LR004 for binding to the EGFR antigen. With an increase in the concentration of unlabeled LR004, the detected radioactivity decreased. The results indicated that ^125^I-LR004 and LR004 bound to the same site on MDA-MB-468 cells, suggesting that the high affinity of LR004 toward EGFR antigen was not affected by ^125^I-labeling.

### 2.7. Biodistribution in Xenograft Mice

**Figure 5:**
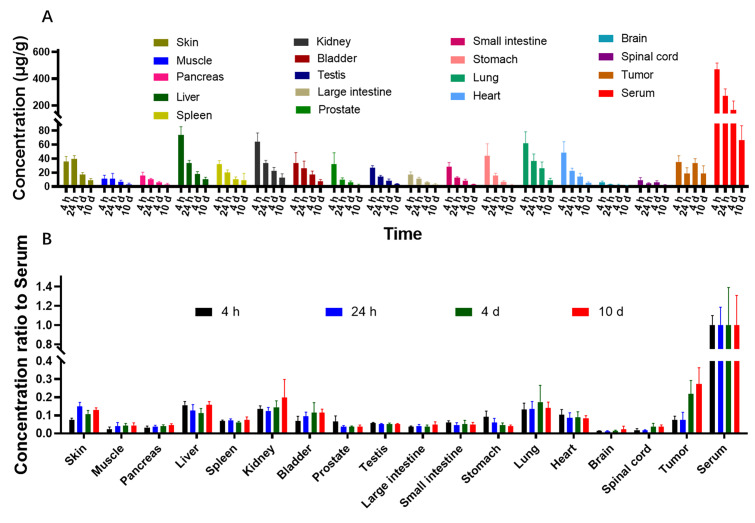
Biodistribution of ^125^I-LR004 in xenograft mice. (**A**) ^125^I-LR004 in tissues at 4 h, 24 h, 4 d, and 10 d following the administration of 60 mg/kg to tumor-bearing mice (n = 6); (**B**) ^125^I-LR004 level in tissues relative to that in serum at 4 h, 24 h, 4 d, 10 d at a dose of 60 mg/kg in tumor-bearing mice (n = 6).

The concentrations of LR004 in tissues and body fluids were determined by ^125^I isotope labeling combined with TCA protein precipitation. Quantitative data in tissues harvested from tumor-bearing mice were obtained, and the concentration of ^125^I-LR004 in tissues is shown in Figure 5A. ^125^I-LR004 exhibited higher uptake in highly perfused organs (e.g., heart, liver, lung, kidney) compared with poorly perfused organs (e.g., brain, muscle). The concentration of ^125^I-LR004 in normal tissues exhibited a gradual decline from 4 h to 10 d, whereas the concentration in tumors remained stable. Figure 5B shows the concentration of ^125^I-LR004 in tissues compared with that in serum. From 4 d to 10 d, ^125^I-LR004 in tumors relative to serum was significantly higher compared with that in the highly perfused organs, indicating a pronounced tumor target tendency of ^125^I-LR004. The prominent radioactivity accumulation in the liver, particularly at 4 h postadministration, was the result of hepatic clearance of the antibody-based tracer. 

### 2.8. Autoradiography Study in Xenograft Mice

**Figure 6:**
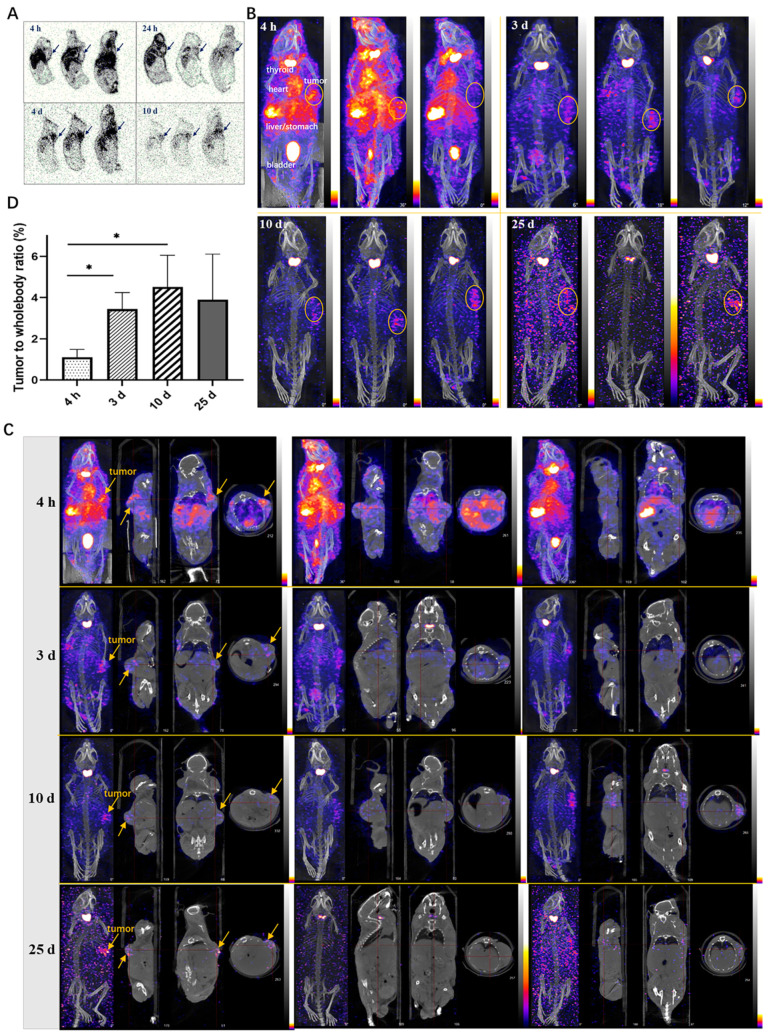
Tumor-targeting of ^125^I-LR004 in xenograft mice through Autoradiography and NanoSPECT/CT imaging. (**A**) Autoradiography of ^125^I-LR004 at a dose of 60 mg/kg for 4 h, 24 h, 4 d, and 10 d in tumor-bearing mice. The black arrows indicate the location of the tumors; (**B**) NanoSPECT/CT of ^125^I-LR004 in tumor-bearing mice at 4 h, 3 d, 10 d, and 25 d postadministration (n = 3). The tumor regions are marked with yellow ellipses; (**C**) In vivo tumor targeting of ^125^I-LR004 assessed via NanoSPECT/CT. The tumor regions are marked with yellow arrows; (**D**) The tumor-to-whole body ratios of radioactivity at 4 h, 3 d, 10 d, and 25 d postadministration of ^125^I-LR004 (* *p* < 0.05).

The autoradiography results of frozen tissues in tumor-bearing mice revealed the widespread distribution of ^125^I-LR004 across various tissues (Figure 6A). The radiation exposure in blood flow-rich tissue was significantly higher than that in other tissues. Over time, the concentration of ^125^I-LR004 in the serum gradually decreased, and radiation exposure levels in tissues also diminished; however, the radioactivity in tumor tissues increased. Marked aggregation of radiation in tumor tissues (black arrow) was observed at 24 h, 4 d, and 10 d postadministration, highlighting the targeted distribution of ^125^I-LR004 in tumors.

### 2.9. NanoSPECT/CT in Xenograft Mice

NanoSPECT/CT results of ^125^I-LR004 are presented in Figure 6B. A significant radioactive exposure of ^125^I-LR004 was observed in the highly vascularized tissues (stomach, bladder, heart, and liver) as early as 4 h postadministration, and a high distribution of ^125^I-LR004 was also observed in the tumors. Radiation exposure levels in the whole body decreased with time, particularly in the highly radioactive organs (e.g., liver, lung, and kidney) during the initial time points. As shown in Figure 6C, tumor tissues with significantly higher drug concentrations showed a marked accumulation of radioactivity compared with the other tissues on Day 3. This high radioactivity level persisted until Day 10, consistent with the findings from our biodistribution study. Although the residual radioactivity in the whole body was considerably low at day 25, hotpots in the tumors were still evident and exhibited a clear contrast with surrounding tissues and background. From 4 h to 10 d postadministration, the prolonged retention of ^125^I-LR004 in tumors combined with its rapid clearance from other tissues resulted in favorable tumor-to-whole body ratios (Figure 6D). The residual radioactivity in the whole body was considerably low at 25 days postadministration, whereas the hot spots within tumors in most mice were still visible with an excellent target–background contrast. These results indicated that ^125^I-LR004 exhibits good tumor targeting capability with a prolonged half-life.

## 3. Discussion

This is the first study to investigate the in vivo PK properties and biodistribution profile of a novel anti-EGFR monoclonal antibody (LR004). Our findings demonstrated that LR004 can specifically bind to the EGFR antigen with appropriate antigen binding activity. This characteristic limits any side effects associated with higher affinity while mitigating the poor therapeutic effects resulting from lower affinity. Detailed studies on the PK of therapeutic antibodies are essential for their continued development. We revealed that LR004 exhibits a prolonged elimination half-life and nonlinear PK characteristics within a dose range of 6–54 mg/kg in rhesus monkeys. According to literature reports [17], cynomolgus monkeys administered a single intravenous dose of 5 mg/kg cetuximab exhibited an accelerated elimination of the drug from plasma at 3–10 days post-administration, with plasma cetuximab concentration falling below the lower limit of quantitation (LLOQ) within 10–14 days. In our PK experiment, rhesus monkeys received a low intravenous dose of LR004 at 6 mg/kg, and similarly, blood concentration started to decline significantly after 3 days and became undetectable after 240 h (10 days). This observation closely resembles the behavior observed with cetuximab. In addition, based on the PK parameters determined through non-compartmental pharmacokinetics analysis, cetuximab exhibited a CL of 0.87 mL/h/kg and a t_1/2_ of 36 h. Similarly, LR004 demonstrated comparable values with a CL of 0.86 ± 0.18 mL/h/kg and a t_1/2_ of 51.60 ± 8.36 h. Furthermore, LR004 displayed nonlinear pharmacokinetic characteristics in monkeys that were consistent with those previously reported for cetuximab [17,18]. Nonlinear PK phenomenon may be associated with target-mediated drug disposition, which is more frequently observed in monoclonal antibody drugs, especially for those targeting membrane receptors [19]. There was no statistically significant plasma accumulation of LR004 after four consecutive intravenous infusions at a dose of 18 mg/kg, indicating that LR004 may have a better safety profile. 

Radionuclide tracing technology is an important means of preclinical and clinical pharmaceutical research. The elimination half-life (t_1/2_) of LR004 at a medium dose in rhesus monkeys was >100 h. ^125^I exhibits a high labeling efficiency and prolonged physical half-life (60 days), which corresponds to the long biological half-life of LR004 in the blood. The classical biodistribution study of ^125^I-LR004 revealed higher concentrations and rapid elimination in highly perfused organs, such as the heart, liver, lung, and kidney, which probably reflects hepatic metabolism and renal excretion. In tumor tissue, the levels of ^125^I-LR004 remained relatively constant, with a peak concentration occurring as early as 4 h and sustained concentrations up to 10 days, indicating the targeted distribution tendency in EGFR-positive tumors. The enhanced visualization of the biodistribution tumor targeting properties of ^125^I-LR004 was further confirmed through autoradiography and NanoSPECT/CT imaging findings.

^125^I-LR004 exhibited reduced radioactivity in poorly perfused organs (e.g., brain, spinal cord, muscle), which may contribute to mitigating potential side effects in these tissues. The accumulation of radioactivity in the thyroid can be attributed to the in vivo deiodination and specific uptake of iodine by the thyroid glands. To accurately reflect real physiological conditions, we did not preblock the thyroid gland with iodine in our experiment. In this study, we employed the conventional iodogen method to label ^125^I onto LR004. In future investigations, we could potentially explore alternative radiolabeling methods such as Bolton–Hunter reagent [20] or N-succinimidyl-para-iodobenzoate (SPIB) [21]. As previously reported, EGFR inhibition may cause lesions in the upper torso, face, neck, and scalp of >50% of the patients [22,23]. Consistent with these reports, our biodistribution results showed relatively high radioactivity in the skin. These factors should be taken into consideration for future studies.

LR004 has only human glycan structure, which can reduce the heterogeneity of the antibody and renders it more suitable for future clinical application in humans. In preclinical studies of antibodies, the evaluation of PK and biodistribution in suitable animal models is a central consideration. We performed PK studies of LR004 in monkeys because it is generally considered the most relevant species for predicting the human PK of monoclonal antibodies. This approach can provide more compelling predictive data for appropriate dosage and timing for future clinical application. For biodistribution studies, we utilized the human tumor cell-xenografted mouse models and employed the noninvasive SPECT/CT imaging techniques, which may also be closer to the clinical investigations. Although the data obtained from animal models differ from that collected in human subjects, this data can still provide valuable insights for subsequent human experiments involving LR004. Overall, LR004 shows promising preclinical PK and biodistribution characteristics, supporting its potential as an effective antibody for treating colorectal cancer.

## 4. Materials and Methods

### 4.1. Reagents, Cells, and Animal Models

All the reagents used were of analytical grade. LR004 was generated by Synermore Biologics Co., Ltd., Suzhou, China (protein content: 5 mg/mL, storage: −20 °C). ^125^I was purchased from PerkinElmer Co., Ltd., Shanghai, China. with a radiation radionuclide purity of 99.90%. Trichloroacetic Acid (TCA) was purchased from Sinopharm Chemical Reagent Co., Ltd, Beijing, China. The BCA Protein assay kit (Lot. 162495,) was purchased from ThermoFisher Co., Ltd., Shanghai, China. The TMB Kit (Lot. 101095992) was purchased from Sigma-Aldrich Co., Ltd., Shanghai, China. The human breast cancer MDA-MB-468 cells and human epidermal carcinoma A431 cells were obtained from JOINN Laboratory (Beijing). Human epidermal carcinoma A431 cells were subcutaneously implanted into BALB/c nude mice (40 males, body weight: 20–30 g) at JOINN Laboratories. Rhesus monkeys with a body weight of 4.5 ± 1.0 kg were purchased from the Experimental Animal Center of Academy of Military Medical Sciences and raised in the Experimental Animal Center of Academy of Military Medical Sciences (Beijing). All animal experiments were performed under standardized conditions, with an ambient temperature of 22 °C ± 2 °C, relative air humidity 40–70%, a 12-h light/12-h dark cycle, with free access to water and food. All animal procedures were performed in accordance with the ARRIVE guidelines and approved and reviewed by the Beijing Institute of Radiation Medicine (Beijing, China, IACUC-DWZX-2021-735). 

### 4.2. BIAcore Analysis of Antibody Affinity and Kinetics

BIAcore analysis was performed as previously described with minor modifications [24]. The affinity of LR004 and cetuximab toward the EGFR antigen was assessed separately under identical experimental conditions. LR004 or cetuximab was diluted to 5.0 μg/mL with HEPES buffered saline supplemented with EDTA and Surfactant P20 (HBS-EP), whereas recombinant human EGFR was diluted to 8, 16, 32, 64, and 128 nM with HBS-EP buffer. A consistent concentration of 32 nM was established as the repeat concentration. A regeneration solution consisting of 10 mM glycine–HCl at pH 2.5 was used. The run program in the WorkFlow wizard mode was employed, and multiple cycles were selected. The resulting data were fitted using BIAcore X100 analysis software(version 1.0, GE Healthcare) by plotting time on the x-axis and signal value on the y-axis to determine the kinetic constants.

### 4.3. PK of LR004 in Rhesus Monkeys

A total of 24 rhesus monkeys (12 males and 12 females) weighing 4.5 ± 1.0 kg were randomly divided into 4 groups, with 6 animals per group. Three groups were administered with a single intravenous infusion at different dose levels (low dose: 6 mg/kg, medium dose: 18 mg/kg, and high dose: 54 mg/kg), and the fourth group received a series of doses with a medium dose (18 mg/kg) of LR004 for four intravenous infusions. For the single dose groups, blood samples were collected at 0 (predose), 20 min, 40 min, 60 min (at the end of intravenous infusion), 1 h, 2 h, 4 h, 8 h, 12 h, 24 h, 2 days (d), 4 d, 6 d, 8 d, 10 d, 12 d, 14 d, 16 d, 18 d, 20 d, 22 d, 24 d, 26 d, 28 d, and 30 d postadministration. For the group receiving a series of doses, blood sampling for the first and fourth time postadministration was performed according to the abovementioned method, and sampling for the second and third time was performed at 7 days postadministration. All blood samples were coagulated for 30 min at room temperature, after which the serum was harvested and stored at −20 °C until analysis. Serum samples were analyzed via enzyme-linked immunosorbent assay (ELISA), and serum concentration–time profiles were used to estimate the PK parameters via noncompartmental analysis (WinNonlin, version 5.2.1; Pharsight Corporation, Mountain View, CA, USA).

### 4.4. Iodination and Purification of LR004

LR004 was labeled using the iodogen method [25,26]. Briefly, 1 mg of iodogen (Sigma-Aldrich Co., Ltd., Shanghai, China) was dissolved in 0.5 mL of chloroform, and the mixture was added to the bottom of a tube and dried under nitrogen. Then, 1 mL of recombinant anti-EGFR monoclonal antibody (LR004) and 6 mCi of Na^125^I were added. The mixture was incubated for 6 min at 22 ± 2 °C under shaking conditions to complete the reaction. The mixture was purified on a Sephacryl G-50 column pre-equilibrated with phosphate-buffered saline (PBS), and the intelligent calibration instrument was used to determine the γ radiation of the eluant.

The radiochemical purity of ^125^I-labeled LR004 was determined by high performance liquid chromatography (SHPLC) (SHIMADZU LC-20 AT) with a TSK-GEL G300SWXL gel filtration column (300 mm × 7.8 mm, 5 mm) and an eluent of 0.05 mol/L phosphate buffer, pH 7.4, at a flow rate of 1.0 mL/min. Each tube of eluent was collected per minute with a fraction collector and γ radioactivity was detected. The radioactive peak area percentage with the same chromatographic profile as the non-labeled LR004 standard was calculated.

### 4.5. In Vitro Inhibition of Tumor Cells

The inhibitory effect of the radiotracer in tumor cells was assessed through CCK-8 cell viability analysis [27]. MDA-MB-468 cells were cultured in DMEM/F12K (1:1) containing 10% fetal bovine serum and 1% penicillin and streptomycin at 37 °C in a humidified incubator with 5% CO_2_. Cells in the logarithmic growth phase were diluted with detection medium to a density of 5 × 10^4^ cells/mL, 100 μL were added to each well, and incubated at 37 °C with 5% CO_2_ for 1 h. LR004 and ^125^I-LR004 were separately diluted to 50, 16.67, 5.56, 1.85, 0.62, 0.21, 0.07, and 0.023 μg/mL using DMEM/F12 medium without bovine serum. After incubating at 37 °C for 24 h, medium containing 5% fetal bovine serum was added, and the cells were incubated for 96 h. CCK-8 solution was added followed by measurement of optical density (OD) values at a wavelength of 450 nm using a microplate reader. The binding rate curve and EC_50_ (amount of antibody for 50% binding) values were determined using GraphPad Prism 8.0.1 (GraphPad Software, San Diego, CA, USA).

### 4.6. In Vitro Binding Activities with EGFR Antigen

The antigen binding activities of LR004 before and after labeling were assessed by ELISA. Specifically, the wells were coated with 0.04 μg/mL EGFR antigen overnight at 4 °C. Subsequently, the plates were washed and blocked with 2% BSA for 2 h at 37 °C. Radiolabeled and non-labeled LR004 were gradually diluted (15, 10, 5, 2, 1, 0.5, 0.2, 0.1, and 0.05 ng/mL) and added to the wells in duplicate. After incubation for 2 h at 37 °C, the plates were washed and anti-human Fc-HRP was added and incubated for 1 h at 37 °C. After washing, 100 μL of TMB substrate working solution was added to each well and the reaction was terminated by adding 2 mol/mL H_2_SO_4_. The OD was measured using a microplate reader at 450 nm and 630 nm.

### 4.7. Specific Binding Affinity of ^125^I-Labeled LR004 toward MDA-MB-468 Cells

Unlabeled LR004 was initially diluted into nine concentration gradients (0.5, 1, 5, 10, 50, 100, 500, 1000, and 5000 μg/mL), and 50 µL of the solution was added to each triplicate tube. Subsequently, ^125^I-labeled LR004 was diluted to a concentration of 50 µg/mL, and 100 µL of the solution was added to each tube. MDA-MB-468 cells at a density of 1 × 10^5^/mL (50 µL) in DMEM/F12 medium (without bovine serum) were added to the solution while stirring gently to prevent cell precipitation. The samples were incubated at 37 °C for 3 h, followed by an additional incubation at 4 °C for 30 min. All samples were washed thrice with PBS. Cell-bound ^125^I-LR004 was separated via centrifugation and quantified via γ counter.

### 4.8. Mouse Tumor Xenografts

Mice bearing human epidermal carcinoma A431 tumors were euthanized via cervical dislocation after isoflurane anesthesia. Tumors were removed under aseptic conditions, sliced into small pieces with a volume of 3 mm^3^, and subsequently inoculated into male nude mice in the right axillary subcutaneous region near the dorsal region. The tumors were allowed to grow for 4 weeks until reaching an approximate volume of 300 mm^3^. The tumor-bearing mice were then used for in vivo studies.

### 4.9. Biodistribution Study in Xenograft Mice

Biodistribution study was conducted following the established methodology described in the literature [28,29]. Four groups of A431 xenograft tumor-bearing mice (n = 6) were intravenously injected with ^125^I-LR004 at a dose of 60 mg/kg and a radiation dosage of 10,641 KBq/kg. The mice were euthanized at 4 h, 24 h, 4 d, and 10 d postadministration. Tissue samples including the skin, muscle, pancreas, liver, spleen, kidney, bladder, prostate, testis, large intestine, small intestine, stomach, lung, heart, brain, spinal cord, tumor, and serum were collected and weighed. Equal amounts of saline and 30% TCA were added to the tissue samples for protein precipitation. After centrifugation at 5000 rpm for 5 min, the supernatant was discarded, and radioactivity in the precipitate per gram of tissue was quantified using a γ counter (Wallac 1470, PerkinElmer^TM^, Waltham, MA, USA). The biodistribution data are expressed as the concentration of ^125^I-LR004 in tissues and the ratio of ^125^I-LR004 in tissues relative to that in the serum.

### 4.10. Autoradiography in Xenograft Mice

Autoradiography was performed as previously described with minor modifications [30]. Tumor-bearing mice were administered ^125^I-LR004 via tail vein injection at a dose of 60 mg/kg and a radiation dosage of 10,641 kBq/kg. Mice were euthanized at 4 h, 24 h, 4 d, and 10 d postadministration. Carcasses were embedded in 2.5% carboxymethyl cellulose and frozen at −20 °C. After being completely frozen, mice were positioned on their left side and fixed onto a slice airborne platform. Overall, 40-µm thick sections were collected at three different positions: tumor sections, right-side limbs, and spinal position. The dried sections were exposed to a phosphor imaging plate for 3 h and scanned using a bio-imaging analyzer (Cyclone Plus, PerkinElmer^TM^) to obtain images.

### 4.11. NanoSPECT/CT Imaging in Xenograft Mice

Twelve tumor-bearing mice (n = 3) were injected with ^125^I-LR004 at a dose of 50 mg/kg and a radiation dose of 199,633 kBq/kg. NanoSPECT/CT scans were performed at 4 h, 3 d, 10 d, and 25 d after administration using a NanoSPECT/CT scanner (Bioscan Inc., Poway, CA, USA). The mice were anesthetized with 3% pentobarbital (50 mg/kg body weight) and placed in a prone position on an imaging platform for each scan. Whole body radionuclide images were reconstructed using an iterative ordered subset expectation maximization two-dimensional algorithm and fused with CT images using the integrated CT system [31,32]. For semi-quantitative analysis, the tumor-to-whole body ratios of radioactivity were determined by drawing a region of interest around the tumors.

### 4.12. Statistical Analysis

All data are presented as means ± standard deviation (SD). GraphPad Prism 8.0.1 (GraphPad Software, San Diego, CA, USA) was used for graph generation and statistical analysis. Significance was evaluated using a Student’s *t*-test, and *p* < 0.05 was considered statistically significant.

## 5. Conclusions

We conducted a comprehensive preclinical study to assess the PK properties and biodistribution of LR004, a novel antitumor antibody. LR004 exhibits specific binding to the EGFR antigen with appropriate antigen binding activity. The elimination half-life (t_1/2_) of LR004 at a medium dose in rhesus monkeys exceeded 100 h, and there was no statistically significant plasma accumulation after four consecutive intravenous infusions. ^125^I-LR004 displayed good tumor targeting capabilities in tumor-bearing BALB/c nude mouse models. Prolonged retention properties of ^125^I-LR004 in tumors were observed at 4 and 10 days. The overall preclinical PK and biodistribution profiles strongly support the potential clinical development of LR004 as an effective antibody for treating colorectal cancer.

## Figures and Tables

**Table 1:** Mean pharmacokinetic parameters of LR004 following intravenous infusion administration to rhesus monkeys at doses of 6, 18, and 54 mg/kg.

Parameters	6 mg/kg	18 mg/kg	54 mg/kg
C_max_ (µg/mL)	148.38 ± 25.66	352.71 ± 40.40	985.25 ± 31.68
T_max_ (h)	1.00 ± 0.00	1.00 ± 0.00	1.00 ± 0.00
AUC_last_ (mg·h/L)	6900.24 ± 1243.11	25,398.82 ± 4774.38	72,301.13 ± 16,411.89
MRT (h)	62.23 ± 4.98	103.93 ± 10.35	127.46 ± 23.48
CL [mL/(h·kg)]	0.86 ± 0.18	0.72 ± 0.12	0.79 ± 0.22
Vd (mL/kg)	62.56 ± 8.78	106.51 ± 9.37	139.83 ± 20.81
t_1/2_ (h)	51.60 ± 8.36	104.62 ± 13.42	130.72 ± 39.57

C_max_: maximum observed serum concentration; T_max_: time to peak concentration; AUC_last_: area under the concentration–time curve from the time of first dosing to last dosing; MRT: mean residence time; CL: clearance rates; Vd: apparent volume of distribution; t_1/2_: terminal elimination half-life.

## Data Availability

All research data can be found within this publication.
